# A Bioinspired Flexible Pressure Sensor with Rigid–Flexible Coupling Featuring Simple Fabrication, Wide Pressure Range, and High Sensitivity

**DOI:** 10.3390/biomimetics11080594

**Published:** 2026-08-20

**Authors:** Zhen Tang, Xingze Chen, Xin Wang, Yangfan Yang, Shanhong Tang, Linpeng Liu

**Affiliations:** 1 School of Automotive and Energy, Xiangtan Institute of Technology, Xiangtan 411100, China; 15273271107@hngeelyedu.cn (Z.T.); 13549640564@hngeelyedu.cn (Y.Y.); 18773219602@hngeelyedu.cn (S.T.); 2 State Key Laboratory of Precision Manufacturing for Extreme Service Performance, College of Mechanical and Electrical Engineering, Central South University, Changsha 410083, China; 253711063@csu.edu.cn (X.C.); 253711004@csu.edu.cn (X.W.)

**Keywords:** pressure sensor, minimum response, rigid-flexible coupling, environmental sensing

## Abstract

Flexible pressure sensors with porous structures are essential for wearable electronics and robotic perception. However, traditional porous flexible sensors suffer from poor stability and long recovery times. To address these challenges, a strategy integrating bionic architectures inspired by the rigid–flexible coupling structure of *Bambusa textilis* and the micro-protrusion structure of *Salvia plebeia* R.Br. is proposed. An aluminum sheet serves as the support layer, and the sensing layer is prepared through mold replication, material impregnation, and layer-by-layer assembly, offering a simple and scalable fabrication route. The sensor exhibits a broad effective pressure range of 0–31.5 kPa, with a minimum resolvable force of 0.2 N, and within its operating range (0–23.5 kPa), the relationship between resistance and pressure exhibits a trend that can be fitted to a quadratic term, with a coefficient of determination of 0.9986. It achieves a minimum response time of 350 ms and maintains stable signals under dynamic loading at different frequencies, indicating reliable detection of low-frequency weak pressures. After 5000 loading–unloading cycles, the device shows no obvious performance degradation. When mounted on a robotic foot, the sensor successfully distinguishes land, sponge, sand, and pebbles surfaces, demonstrating its potential for intelligent environmental perception.

## 1. Introduction

The rapid evolution of large-scale integrated circuit technology and the continuous expansion of functional material systems have significantly advanced flexible electronics. Among these developments, wearable electronic devices that combine excellent mechanical flexibility with good biocompatibility are increasingly entering practical use [1,2,3,4,5]. As a key component in this field, flexible pressure sensors offer distinct advantages such as conformal contact with curved surfaces and strong adaptability to deformation. They therefore hold considerable promise in applications including real-time pressure monitoring, health diagnostics, and motion capture [6,7,8,9,10,11,12,13].

At present, flexible pressure sensors are primarily based on piezoresistive [14], capacitive [15], or piezoelectric [16,17] transduction mechanisms. Among these, piezoresistive sensors are the most widely researched and applied, owing to their broad pressure detection range, simple operating principle, and straightforward fabrication process. To further improve the mechanical and electrical performance of piezoresistive devices, various micro and nanostructures [18,19,20,21,22] such as porous architectures [23,24], surface protrusions, and microcracks have been progressively incorporated into sensor designs [25,26,27,28,29]. In parallel, rigid–flexible coupled structures have emerged as an important design strategy [30,31,32,33,34], combining the structural stability, mechanical strength, and precision of rigid materials with the bendability, stretchability, and shape adaptiveness of flexible layers. Introducing such coupling into pressure sensors can substantially enhance their mechanical compliance and conformability while also improving sensitivity and output signal quality. Despite these advances, constructions based on porous conductive sponges and rigid substrates though suitable for many engineering scenarios still exhibit inherent limitations. The discrete point–contact interfaces between the porous foam and the electrodes tend to fluctuate under cyclic loading, leading to degraded signal stability, reduced fidelity, and noticeable baseline drift over prolonged operation. These issues call for further structural refinement. Numerous studies have confirmed that integrating multiple micro and nanostructures into a single sensor can effectively overcome the performance bottlenecks of single-structure designs. For instance, combining a porous elastic matrix with micro-protruded surface features can improve the interfacial contact state, simultaneously enhancing both sensitivity and linearity. A well-designed multistructure coupling not only optimizes overall performance metrics, but also simplifies fabrication and reduces cost, offering both scientific and practical value [35,36,37]. Moreover, Liu et al. proposed a coupling scheme of porous matrix and surface micro-protrusion microstructure, which achieves prominent improvement in sensing stability, and its anti-fluctuation performance of signal is around 12 times that of sensors without micro-protrusions [38]. Zhang et al. constructed a flexible porous sensing system with porous pyramid microstructures, which realizes the simultaneous enhancement of sensitivity while cutting down the fabrication cost of devices [39]. Wang et al. designed a sandwich porous microstructure composed of porous nanofiber mat and a microcone array film, which simultaneously achieves remarkable optimization on both the sensitivity and effective pressure detection range of the sensor [40]. In the rigid–flexible coupling system proposed in this work, all structural components work synergistically to optimize the overall sensing performance. The surface micro-protrusions improve low-pressure sensitivity through the tip stress concentration effect and enhance the stability of output electrical signals. The three-dimensional interconnected pores of the porous conductive sponge collapse progressively with rising pressure, which effectively extends the detection range. The rigid aluminum sheet constrains the viscoelastic creep of the flexible matrix, accelerates elastic rebound after unloading to shorten the response and recovery time, and alleviates baseline drift during long-term operation.

To achieve both fast response and high stability, this paper proposes a multistructure coupled pressure sensor based on the rigid–flexible coupling of *Bambusa textilis* and the micro-nano structures of *Salvia plebeia* R.Br. This sensor integrates micro-protrusions, porous sponge, and a rigid aluminum plate into a single rigid–flexible composite. The aluminum plate serves as a rigid backbone, reinforcing the mechanical strength, accelerating deformation recovery, and extending the pressure response range. An insulating adhesive layer between the conductive sponge and the aluminum plate prevents electrical crosstalk, ensuring accurate signal output. Unlike conventional single-form sensors, the device features an innovative overall configuration that further enhances dynamic response and conformal adaptability. The results show that the sensor operates effectively up to 31.5 kPa, covering a broad pressure range, and can reliably resolve minute load variations as small as 0.2 N. Its minimum response time reaches 350 ms, indicating excellent dynamic responsiveness. After 5000 consecutive loading–unloading cycles, the output signal exhibits no significant attenuation or baseline drift, demonstrating outstanding long-term stability. In practical tests, when mounted on a robotic leg, the sensor successfully distinguishes different surfaces with high precision. Collectively, these results confirm that the proposed rigid–flexible coupled pressure sensor holds strong potential for applications in environmental perception and intelligent sensing for rescue and exploration robots.

## 2. Materials and Methods

### 2.1. Materials

Commercial conductive carbon ink was purchased from M&G Chenguang Stationery Co., Ltd., Shanghai, China. Multi-walled carbon nanotubes (MWCNTs) were obtained from Suzhou Tanfeng Graphene Technology Co., Ltd., Suzhou, China. Waterborne polyurethane dispersion was supplied by Zhengmeisu Adhesive Co., Ltd., Guangzhou, China. Sandpaper (60 grit) was purchased from Hubei Yuli Abrasive Belt Group, Xianning, Hubei, China. Melamine foam was acquired from a local market. The aluminum sheets were purchased from Weng Hou Metal Materials Co., Ltd., Hefei, China. PDMS (Sylgard 184) was purchased from Dow Corning Corp, Shanghai, China. Silver paste was supplied by Julong Electronic Technology Co., Ltd., Shenzhen, China.

### 2.2. Preparation of Surface Stochastic Microstructure

Sandpaper is used as the molding template. Sandpaper is selected due to its low cost, wide availability, and tunable surface roughness, which can be easily varied by choosing different grit sizes. The PDMS prepolymer and curing agent were mixed uniformly at a weight ratio of 10:1, then coated onto the surface of sandpaper, cured in an oven at 60 °C for 2 h, and subsequently peeled off to obtain a negative PDMS mold with a micropatterned structure. Subsequently, a mixed solution of multi-walled carbon nanotubes (MWCNTs) and polyurethane (PU) is cast onto this mold. The MWCNT/PU mixture was prepared by mixing MWCNTs with PU at room temperature under mechanical stirring at 1000 rpm for 2 h to ensure homogeneous dispersion. The resulting composite solution was then cast into films and cured in an oven at 60 °C for 4 h to obtain the MWCNT/PU film. The MWCNT/PU blend provides both electrical conductivity and mechanical flexibility, ensuring that the final film can endure bending and stretching without cracking. After curing and demolding, a conductive film with micro-protrusions on one side is obtained, and the protrusion geometry faithfully replicates the inverse features of the sandpaper template.

### 2.3. Assembly of the Flexible Pressure Sensors

First, the sponge was cut into a strip with a size of 80 mm × 20 mm (*l* × *w*). Then, an insulating melamine sponge is immersed in carbon black ink and subjected to ultrasonic treatment for 2 h to ensure thorough infiltration of the ink into the sponge skeleton. The ultrasonic agitation helps disperse the carbon particles uniformly and drives them into the open pores of the sponge, preventing agglomeration and ensuring a homogeneous conductive coating. The sponge is then dried in an oven at 70 °C for 2 h, yielding a conductive sponge that retains its intact porous structure. Afterward, the conductive film is also cut into the same shape. Finally, the aluminum sheet, conductive sponge, microstructured film, and copper electrodes are assembled in sequence. An insulating double-sided adhesive tape is placed between the sponge and the aluminum sheet to prevent electrical contact, which would otherwise cause short-circuiting and distort the measurement signal. At the interfaces among the conductive sponge, the microstructured film, and the copper electrodes, a CNT/PU mixture combined with silver paste is applied to bond and cure the connections, completing the electrode encapsulation. The aluminum sheets were also custom-fabricated with specified thicknesses. For the active functional layer (the MWCNT/PU film), a uniform and consistent thickness was achieved by spin-coating the composite solution at 600 rpm for 45 s.

### 2.4. Characterization

The surface morphologies of the sponges before and after immersion were characterized by scanning electron microscopy (SEM, TES-CAN, Brno, Czech Republic). The surface microstructure morphology of the porous sensor was carefully observed using a super-depth-of-field microscope (VH-5000, Keyence, Foshan Zongben Optical Instrument Co., Ltd., Guangzhou, China). The basic performance test was conducted by applying pressure to the sensor using an electrically linear workbench with positioning control. The resistance of the pressure sensor was measured in real time using a multimeter (DAQ6510, KEITHLEY, Solon, OH, USA). The mechanical force applied to the pressure sensor was measured using a strain gauge (HP—1 kN, Handpi Instrument Co., Ltd., Yueqing, Zhejiang, China).

## 3. Results and Discussion

### 3.1. Structural Design and Sensing Mechanism of the Sensor

**Figure 1:**
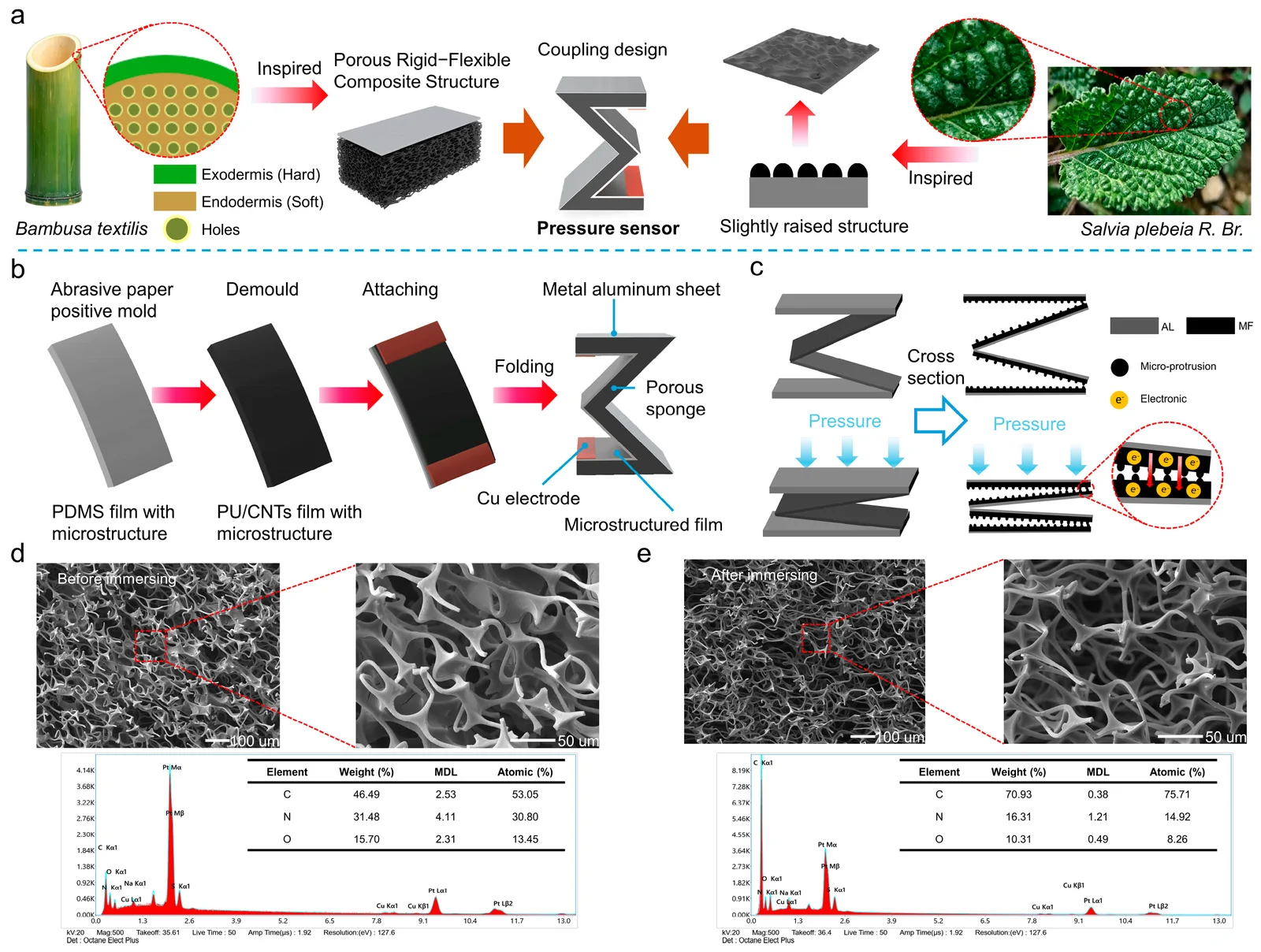
**Fabrication process of the pressure sensor.** (**a**) The design concept of the bioinspired strain sensor based on the strategy of coupling bionics. (**b**) Flowchart of the fabrication process. (**c**) Schematic diagram of the sensing mechanism. (**d**) SEM images and corresponding EDS spectra result before ink immersion. (**e**) SEM images and corresponding EDS spectra result after ink immersion.

This paper presents a flexible pressure sensor featuring multistructure coupling that integrates a rigid–flexible coupled structure with a micro-protrusion structure. The design of this sensor draws inspiration from the gradient layered structure of *Bambusa textilis* and the micro-protrusion structure of *Salvia plebeia* R.Br. The outer bamboo wall is rigid and tough while the inner part is soft and porous; incorporating this characteristic into the sensor design can effectively shorten the response time. Meanwhile, the layered protrusions on the surface of *Salvia plebeia* R.Br. help distribute stress, cushion deformation, and suppress interface slippage. As shown in Figure 1a, the two biomimetic structures are synergistically integrated into a single sensor, enabling the device to deliver both fast response and high stability. The sensor consists of an aluminum foil, high-strength double-sided insulating adhesive, conductive sponge, and a microstructured conductive film featuring an array of micro-protrusions. After assembling the functional components layer by layer and bending them into shape, a W-shaped pressure sensing unit is obtained. The aluminum foil serves as a rigid substrate, which significantly broadens the device’s elastic deformation range and shortens the deformation recovery time. At the same time, by laminating the microstructured film with micro-protrusions onto the conductive sponge, a layered composite W-shaped piezoresistive sensor with an “HCF-CS-RAPS” layered structure was ultimately fabricated. Benefiting from the rigid–flexible coupling architecture, this synergistic combination effectively shortens the viscoelastic relaxation time and significantly alleviates the problem of slow response in traditional porous flexible sensors [41,42,43,44,45,46]. The comparative data are summarized in Figure S1.

The fabrication process is shown in Figure 1b. First, the conductive sponge is prepared by immersing a melamine sponge in carbon black ink, followed by ultrasonic treatment and drying in an oven at 70 °C. This process preserves the sponge’s original porous structure while allowing flexible control over the loading of conductive fillers. The microstructured film was fabricated using sandpaper as a replication template. PDMS was first poured onto the sandpaper template. After curing, it was peeled off to obtain a negative mold. Next, a carbon nanotube/polyurethane mixed slurry was poured into the negative mold. After curing and demolding, a conductive film with micro-protrusions on its surface was obtained. Subsequently, the aluminum substrate, conductive sponge, microstructured film, and electrodes are assembled in sequence. Insulating double-sided tape isolates the conductive sponge from the aluminum foil to prevent electrical signal crosstalk, and a carbon nanotube/polyurethane–silver paste composite layer is used for bonding at the interface, which not only reduces interfacial contact resistance but also ensures mechanical adhesion between layers. Using this simple assembly process, an integrated flexible pressure sensor can be fabricated. Figure 1c illustrates the pressure-sensing mechanism. When external pressure is applied to the W-shaped sensing unit, two synergistic piezoresistive effects occur simultaneously. For the conductive sponge, compression brings adjacent dendritic skeletons into contact and narrows the internal gaps, thereby increasing conductive channels and reducing resistance. For the micro-protruded film, macroscopically, pressure enlarges the contact area between conductive layers, especially at the folded creases. Microscopically, the surface protrusions make contact first and expand laterally via the positive Poisson effect as pressure rises, creating more conductive paths and leading to a rapid resistance drop. When the contact area ceases to increase, the sensor enters the saturation stage, accompanied by a decline in sensitivity. Benefiting from the rigid constraint of the aluminum sheet, the deformation of each functional layer remains reversible. Thanks to its unique structural design and W-shaped geometry, the sensor’s response can be divided into three distinct stages. In the initial stage, the external load is primarily borne by the sponge. The deformation of the porous sponge and the collapse of its internal pores dominate the decrease in resistance. This stage provides high sensitivity to weak pressures. As the pressure increases to moderate levels, the film begins to share the load, and the micro-protrusions start to come into contact with one another, further reducing the resistance. This synergistic interaction between the sponge and the film significantly enhances sensitivity in the medium-pressure range. In the final stage, both the sponge pores and the film are almost completely compacted. Although the contact area at the film–sponge interface continues to expand, the rate of this expansion has slowed considerably, resulting in only a gradual and slow further decrease in resistance. This three-stage behavior was specifically designed to expand the effective pressure range while maintaining a smooth and predictable resistance–pressure relationship, which will be quantitatively analyzed in subsequent sections. Figure 1d and Figure 1e show the SEM and EDS spectra images of the sponge before and after immersion in carbon ink, respectively. It can be clearly observed that the carbon content of the melamine increased significantly after the ink immersion treatment.

**Figure 2:**
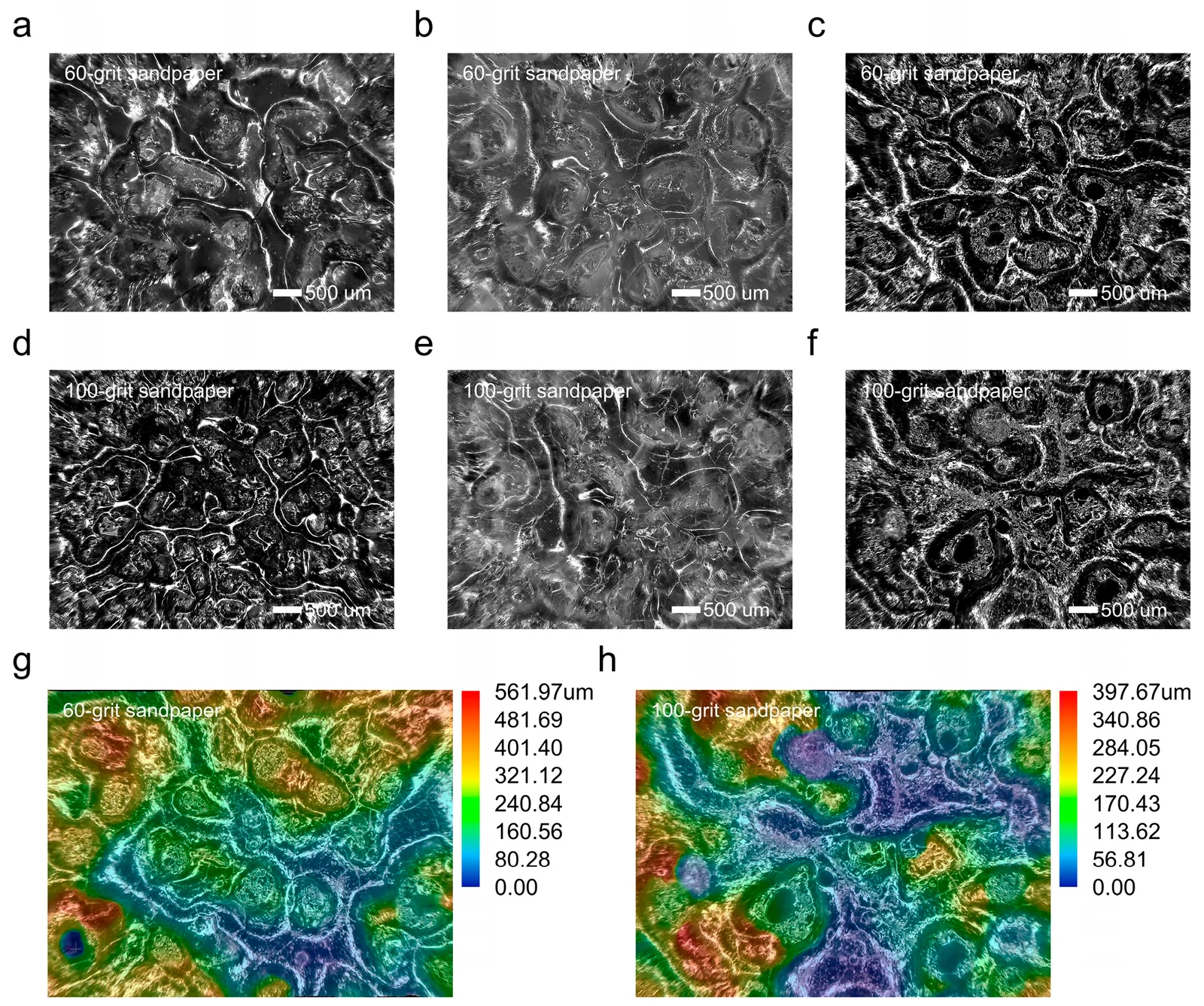
**Surface morphology and sensing mechanism of the sensor.** (**a**–**c**) Digital micrographs of (**a**) the 60-grit sandpaper template, (**b**) the PDMS replica produced via a single molding process (with a base-to-curing-agent weight ratio of 10:1), and (**c**) the resultant microstructured conductive film. (**d**–**f**) Digital micrographs of (**d**) the 100-grit sandpaper template, (**e**) the corresponding PDMS replica (10:1 ratio), and (**f**) the microstructured conductive film. (**g**) Three-dimensional profilometric image of the microstructured film fabricated by secondary replica molding from the 60-grit sandpaper template. (**h**) Three-dimensional profilometric image of the microstructured film fabricated by secondary replica molding from the 100-grit sandpaper template.

To evaluate the fidelity of the biomimetic microstructure replication via the molding process, the surface morphologies of the original sandpapers (60 grit and 100 grit) are compared, the PDMS negative mold is obtained from the first replication, and the final conductive micro-protruded film is examined, as shown in Figure 2a–f. The 60-grit sandpaper features coarser and more pronounced asperities, while the 100-grit one has finer and denser protrusions. The comparison reveals that the microstructure of the twice-replicated film matches the 60-grit sandpaper texture with high precision, confirming that this process can faithfully reproduce the microscopic concave–convex features of the template surface. For the 100-grit template, the replication fidelity is slightly lower, possibly due to the smaller feature sizes approaching the resolution limit of the molding material. Nevertheless, both templates yield well-defined microstructures on the final film, demonstrating the versatility of this simple and low-cost fabrication approach. The high-fidelity replication is crucial because the surface topography directly influences the contact behavior and thus the piezoresistive response of the sensor.

Figure 2g and Figure 2h show three-dimensional profile images of the microstructured conductive film obtained from the 60 grit and 100 grit templates, respectively. These images clearly display the complete topographical contour of the micro-protrusions on the film surface, including their height, lateral dimensions, and spatial distribution. The protrusions from the 60 grit template exhibit larger heights and wider bases, which are expected to enhance the contact area change upon compression, whereas the 100 grit film shows denser but lower protrusions that may offer a more gradual contact progression. Such quantitative topographical information is essential for correlating the structural parameters with the device’s electrical performance.

### 3.2. Electromechanical Response and Dynamic Stability of the Sensor

**Figure 3:**
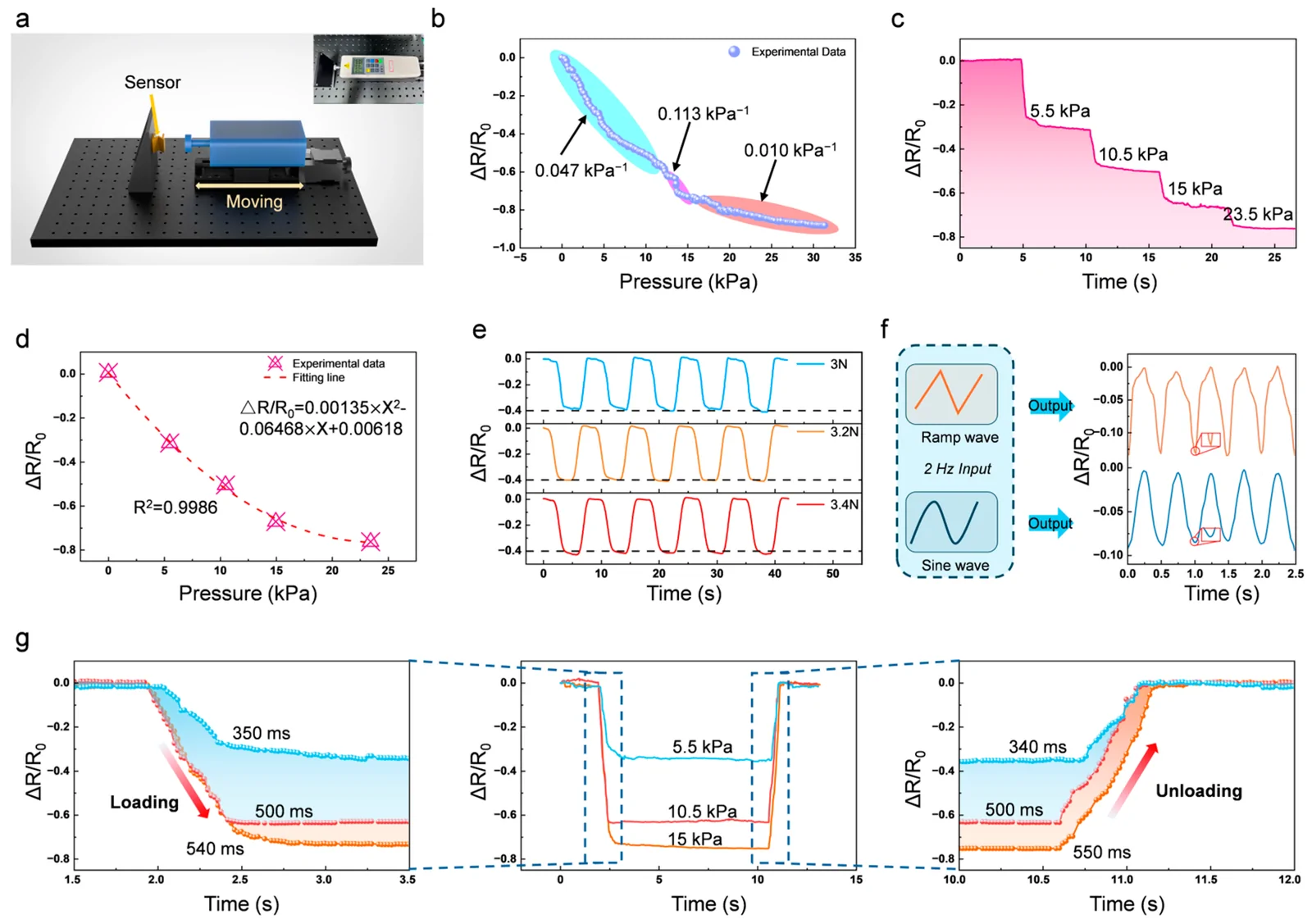
**Performance of the sponge pressure sensor.** (**a**) Schematic diagram of the test setup. (**b**) Relative resistance changes of the sensor under pressures from 0 to 31.5 kPa. (**c**) Resistance changes of the pressure sensor under 20% incremental strain. (**d**) Quadratic polynomial fitting curve of the relative resistance changes under pressure variations. (**e**) Relative resistance changes of the pressure sensor under small pressure variations. (**f**) Ability of the pressure sensor to identify strains with different waveforms. (**g**) Response/Recovery times of the pressure sensor at pressures of 5.5 kPa, 10.5 kPa, and 15 kPa.

Figure 3a shows the schematic of the experimental platform to test the pressure performance of the sensor. This setup enables precise control of applied pressure and strain by regulating the motor displacement and loading rate. Figure 3b depicts the response curve of the sensor under pressure. The curve exhibits a distinct piecewise linear behavior: the sensitivity is 0.047 kPa^−1^ in the low-pressure range of 0–12.5 kPa, it increases markedly to 0.113 kPa^−1^ between 12.5 and 15 kPa, and it decreases to 0.010 kPa^−1^ above 15 kPa. This three-stage response is in good agreement with the previously described piezoresistive mechanism, confirming that the structural design effectively tunes the sensing performance. The transition pressures correspond well to the sequential activation of the sponge deformation, the film protrusion contact, and the final compaction stage, validating our multiscale design rationale.

The sensor’s response to stepwise pressure loads was further investigated. As shown in Figure 3c, as the step pressure increased from 0 to 23.5 kPa, the output signal exhibited a distinct step-down, and the readings from each platform remained stable without any noticeable relaxation. The absence of relaxation indicates that the viscoelastic creep of the sponge and film is minimal, which is advantageous for real-time monitoring applications that require rapid stabilization. As shown in Figure S2, the signal fluctuations at all stages remain within a relatively small range (less than 15%), which effectively verifies the outstanding stability of the device. The curve shown in Figure 3d was obtained by extracting the peak points from each step and fitting them with a quadratic polynomial. The fit yields a coefficient of determination R^2^ = 0.9986, indicating excellent quantitative characteristics and a typical quadratic nonlinear response. This high correlation coefficient demonstrates that the sensor output can be accurately predicted by a simple mathematical model, facilitating calibration and data interpretation in practical systems. The dynamic response to small load variations was also tested by applying a gradient pressure from 3 to 3.4 N. As shown in Figure 3e, the sensor can clearly distinguish force increments as small as 0.2 N, demonstrating excellent force resolution. This level of resolution is sufficient to detect subtle contact forces, such as those caused by a light touch of a finger or the grasping of a small object, making the sensor suitable for delicate manipulation tasks.

To evaluate the sensor’s ability to reproduce different waveform patterns, dynamic excitations with the same frequency (2 Hz) but different waveforms (triangular and sinusoidal) were applied. The results are shown in Figure 3f, where the output waveforms closely follow the input profiles: the triangular wave produces a linear rise–fall response, while the sinusoidal wave produces a smooth, continuous arc with no noticeable distortion or phase lag. In this expanded view, it becomes evident that the sinusoidal waveform is inherently smoother, whereas the triangular waveform displays a more pronounced sharpness at the turning points. This near-perfect waveform tracking confirms that the sensor possesses excellent dynamic tracking capabilities and can faithfully reproduce various forms of periodic pressure stimuli. Response and recovery times are also critical performance parameters; the measurements were performed at three pressure levels (5.5, 10.5, and 15 kPa), and the result is shown in Figure 3g. When the applied pressure is 5.5 kPa, the response and recovery times are 350 ms and 340 ms, respectively, and when the applied pressure grows up to 10.5 kPa, a 500/500 ms of response/recovery time is obtained; the response/recovery time keeps growing once the applied pressure increases to 15 kPa, reaching 540 ms and 550 ms. This trend indicates that both response and recovery times increase with higher applied pressures. The sensor’s response and recovery times rise above 500 ms under high pressure due to the viscoelasticity of the sponge and composite film, as deep compaction prolongs material rebound. Benefiting from the rigid–flexible coupling structure, the aluminum substrate restrains plastic deformation and micro-protrusions form multistage conductive paths. Only local contact separation of microstructures is required to reconstruct conductive networks without full bulk recovery of the sponge, enabling the device to steadily track periodic pressure inputs up to 3 Hz. The prolongation at higher pressures is attributed to the greater deformation of the sponge and film, which requires more time for the viscoelastic materials to return to their original state upon unloading. Nevertheless, even at the highest tested pressure, the recovery time remains below 600 ms, which is adequate for most dynamic sensing applications. These comprehensive characterizations collectively confirm that the sensor possesses a well-balanced combination of high sensitivity, broad range, fast response, and excellent stability, positioning it as a competitive candidate for next-generation flexible pressure sensing systems.

**Figure 4:**
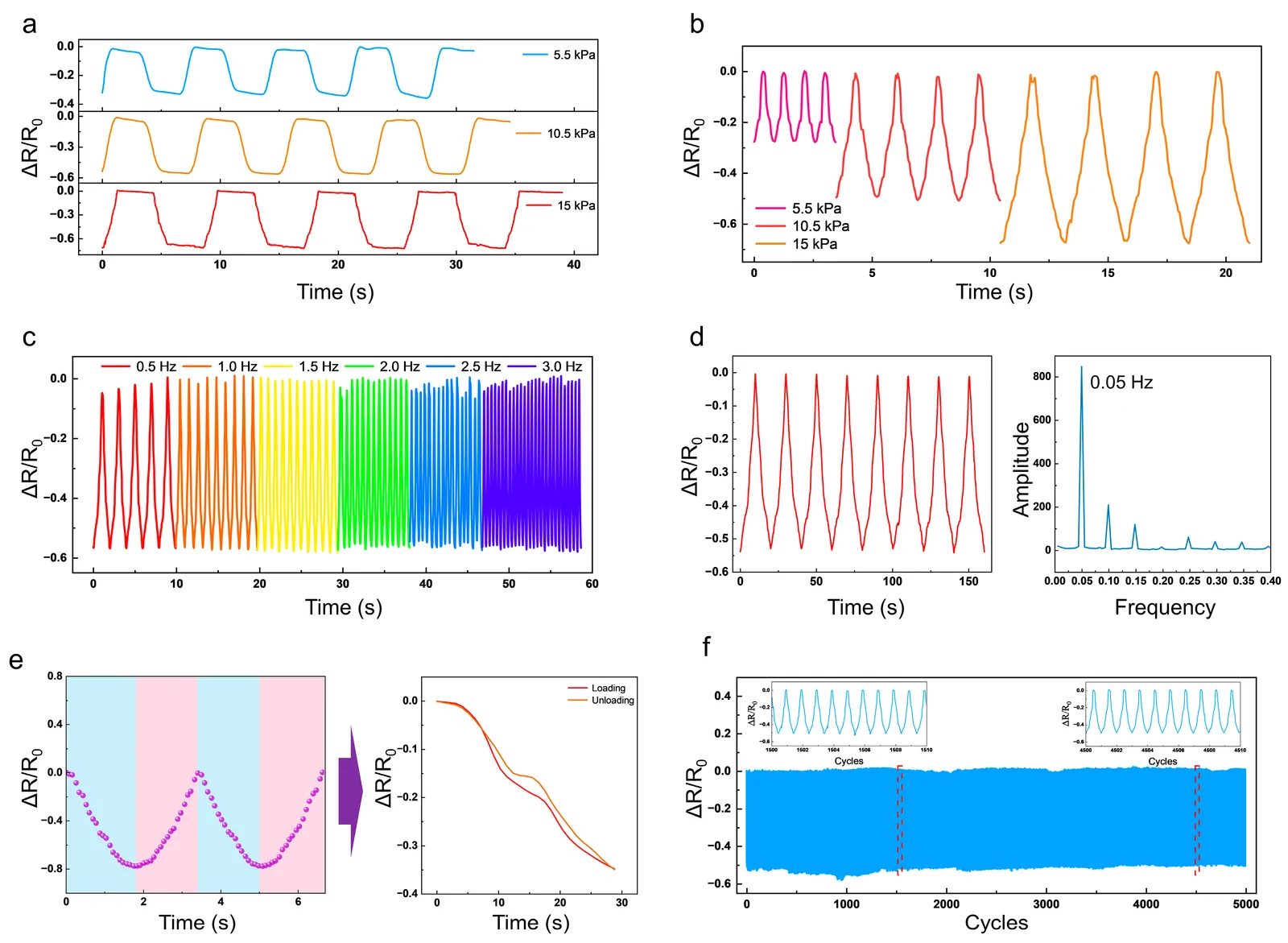
**Dynamic response characteristics of the pressure sensor.** (**a**) Response of the sensor under cyclic dynamic and static loads at 5.5 kPa, 10.5 kPa, and 15 kPa, respectively. (**b**) Relative resistance changes of the pressure sensor under cyclic dynamic loads of 6.5 kPa, 10.5 kPa, and 15 kPa, respectively. (**c**) Response of the sensor under the same load but at different frequencies (0.5 Hz, 1 Hz, 1.5 Hz, 2.0 Hz, 2.5 Hz, 3.0 Hz). (**d**) Resistance changes of the sensor under an external force at 0.05 Hz and the corresponding electrical signal obtained by fast Fourier transform. (**e**) Real-time relative resistance curve during loading and unloading under a fixed pressure of 5.5 kPa. (**f**) Stability test of the sensor under 5000 loading–unloading cycles at a fixed pressure of 10.5 kPa.

Next, to systematically evaluate the dynamic response of the fabricated pressure sensor, cyclic pressure loads of 5.5 kPa, 10.5 kPa, and 15 kPa were applied in ascending order, with loading and unloading each lasting 1 s and a holding time of 4 s for each load level. The corresponding responses are shown in Figure 4a. The sensor exhibits highly reproducible response curves during the loading–hold–unloading cycles, the peak responses increase regularly with increasing pressure, and the signals remain stable without noticeable drift under constant pressure, demonstrating excellent response stability and linearity. Figure 4b shows the response when the hold-pressure phase was subsequently removed and cyclic dynamic pressures of the same amplitude (5.5 kPa, 10.5 kPa, and 15 kPa) were applied. The relative resistance of the sensor rises and falls rapidly as pressure is applied and released, and the magnitude of the response is positively correlated with the pressure level. This synchrony indicates that the sensor can track rapid pressure changes without noticeable delay, which is critical for real-time haptic feedback and vibration sensing. To evaluate the frequency response characteristics, cyclic pressure loads with a fixed amplitude were applied at frequencies ranging from 0.5 to 3.0 Hz, and the results are presented in Figure 4c. The sensor produced stable and consistent response signals across the entire tested frequency range, with no noticeable attenuation in the relative resistance change amplitude, demonstrating excellent signal fidelity and frequency-independent behavior within this bandwidth. This frequency independence arises from the low viscoelasticity of the elastomeric materials and the robust contact mechanics of the micro-protruded film, which together ensure that the sensor does not exhibit rate-dependent hysteresis over this frequency window. Such frequency-independent behavior ensures that the sensor can be reliably applied in scenarios involving complex time-varying loads without introducing artifacts.

Furthermore, to explore the sensor’s capability for detecting weak low-frequency dynamic loads, ultra-low-frequency (0.05 Hz) periodic pressure tests were conducted. As shown in Figure 4d, the sensor still outputs well-defined periodic response waveforms with stable amplitude even under such extremely low excitation. The ability to detect such slow signals is not trivial, as many flexible sensors suffer from baseline drift or poor low-frequency response due to creep or charge leakage. Fast Fourier transform (FFT) analysis reveals that the dominant frequency in the spectrum precisely matches the 0.05 Hz excitation, with no significant spurious peaks or noise, confirming that the device can reliably sense ultra-low-frequency weak pressures, such as slow deformations and gradual environmental loads. This feature is particularly valuable for structural health monitoring where low-frequency dynamic events are prevalent. Hysteresis is a critical indicator for evaluating the reversibility of flexible sensors. To quantitatively evaluate the reversibility of loading and unloading, a single cycle was extracted from a constant-pressure (5.5 kPa) cycling test to compare the changes in relative resistance during the loading and unloading processes, as shown in Figure 4e. The two curves closely overlap with no apparent deviation. The hysteresis error of approximately 9% indicates that the sensor exhibits no significant hysteresis, thereby effectively preventing signal distortion caused by hysteresis. The low hysteresis can be attributed to the elastic recovery of the sponge and the film, as well as the stable interfacial contacts that do not exhibit substantial friction or adhesion differences between loading and unloading paths.

Generally, materials may experience fatigue and plastic deformation after multiple loading/unloading pressure cycles, which can lead to a degradation in the sensing performance of the fabricated sensor. Figure 4f shows the stability of the bio-inspired sensor at a fixed pressure of 10.5 kPa over a total of 5000 loading/unloading cycles. Throughout the entire cycle, the output signal exhibited no noticeable baseline drift or amplitude decay. Enlarged views of the initial and final cycles reveal nearly identical waveform profiles and amplitudes, with no apparent signal drift, confirming excellent mechanical stability and operational reliability over extended periods of use. These results collectively indicate that the sensor maintains stable electromechanical characteristics under long-term cyclic loading, meeting the reliability requirements for practical applications, such as wearable health monitors and robotic tactile sensing systems that demand consistent performance over extended operation times.

### 3.3. Applications of the Sensor in Robotic Terrain Recognition

**Figure 5:**
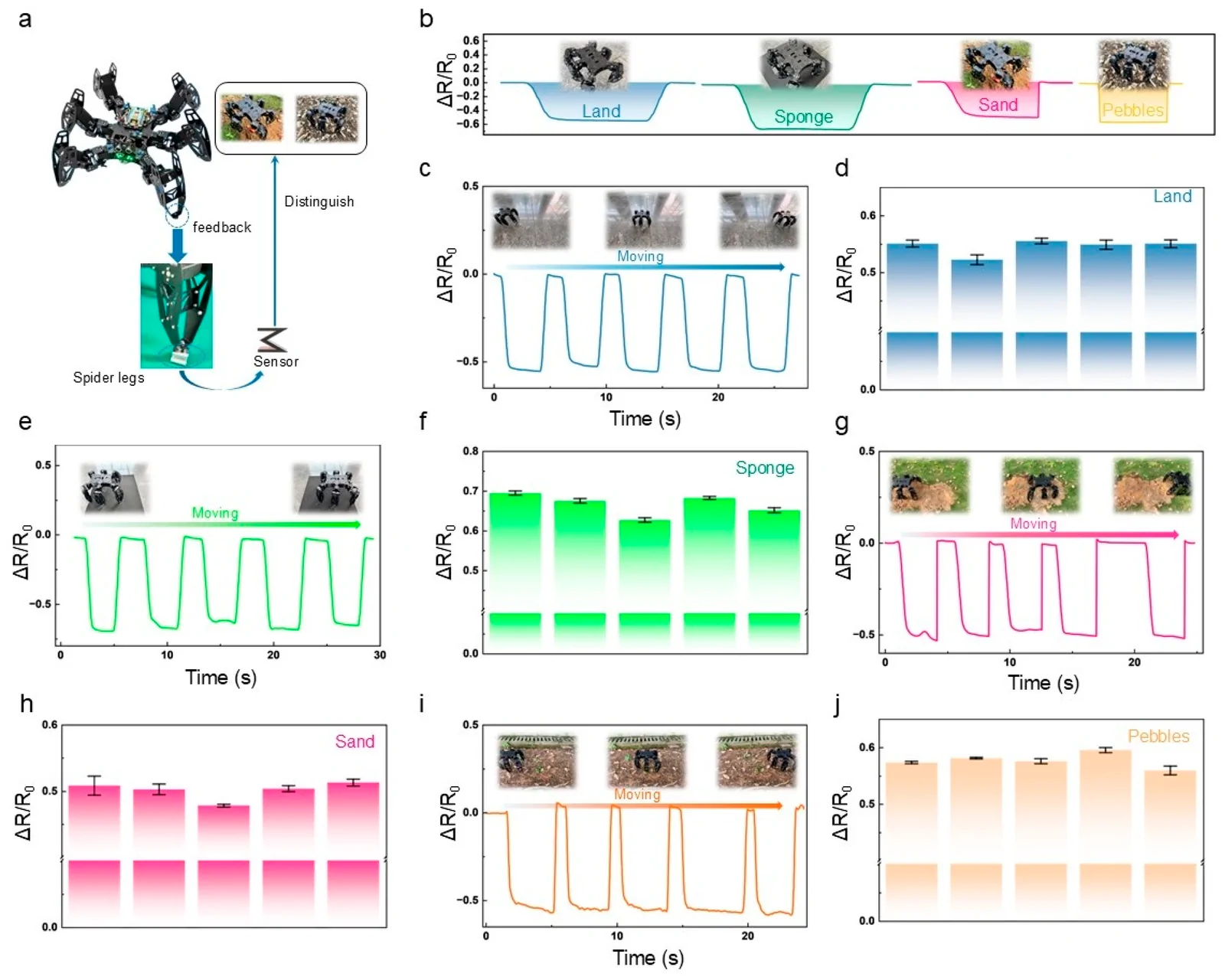
**Application of the pressure sensor in environmental detection.** (**a**) Schematic diagram of the sensor installed on the sole of a six-legged spider robot for environmental detection. (**b**) Relative resistance variation of the pressure sensor when the robot stands on different ground substrates, including land, sponge, sand, and pebbles surfaces. (**c**,**d**) Relative resistance change of the sensor when the robot moves on a land surface. (**e**,**f**) Relative resistance change when moving on a sponge surface. (**g**,**h**) Relative resistance change when moving on a sand surface. (**i**,**j**) Relative resistance change when moving on a pebbles surface.

To demonstrate the practical utility of the sensor, it was integrated with a six-legged robot, and both static and dynamic tests were conducted under various ground conditions. Figure 5a presents that the sensor was mounted on the ventral side of one foot, and the robot was programmed to perform controlled stepping motions to ensure repeatable loading patterns. First, static measurements were carried out by placing the robot (with the sensor mounted on its foot) onto four types of surfaces, which are land, sponge, sand, and pebbles, and then lifting it off after stable loading, while the steady state relative resistance responses were recorded (Figure 5b). These four surfaces were selected to represent typical terrains encountered in indoor and outdoor environments: rigid smooth ground, soft deformable padding, granular loose media, and natural earthy ground. The static test protocol allowed us to isolate the intrinsic response of the sensor to each medium without the confounding effects of dynamic gait variations. The order of the response amplitudes from largest to smallest was: sponge, pebbles, land, sand. This ranking arises from the combined effects of medium stiffness, plastic deformability, and interfacial contact conditions. The highly elastic sponge induces the largest compression of the sensing element, causing the internal conductive pores to close fully and the microstructured film to contact intimately, thereby yielding the highest resistance change. The pebbles offer moderate plastic indentation, resulting in a uniform medium compression and a medium response. The land deforms negligibly, producing only minor surface compression and thus a low response. Loose sand particles tend to slip and disperse the load, establishing only local point contacts with the sensing unit, which gives the weakest effective compression and the lowest response amplitude. These static results not only confirm the sensor’s ability to differentiate materials of contrasting mechanical properties but also establish a baseline reference for interpreting the subsequent dynamic walking data.

Building on these static results, dynamic walking tests were performed on each surface, and the real-time resistance feedback during locomotion was recorded. Figure 5c,d shows the sensor responses recorded while the robot walked on a land surface. The peaks and troughs of the responses exhibited no significant periodic deviations, and the data scatter was minimal. This stability is attributed to the smooth and flat land surface, which ensures uniform and stable foot loads. The highly repeatable waveforms indicate that the sensors can reliably capture periodic stepping motion without being affected by environmental irregularities. Figure 5e,f shows the sensor responses recorded while the robot walked on the sponge surface; the peak-to-valley amplitude fluctuates slightly from one cycle to the next. This is because the internal stiffness of the sponge pad is unevenly distributed, and during continuous walking, the robot’s center of gravity and the instantaneous impact loads on its feet cannot be exactly the same with every step. Nevertheless, the overall waveform pattern remains clear and is consistently distinguishable from that on other surfaces. Figure 5g,h shows the sensor responses recorded while the robot was walking on a sandy surface. The local density of the sand layer varies randomly, and the sand grains come into contact with only discrete areas of the robot, resulting in uneven overall compression. Consequently, the depth of the troughs varies from one gait cycle to the next. Despite this variability, the average response amplitude remains within a characteristic range that clearly distinguishes sand from other terrains. Figure 5i,j illustrates the sensor responses recorded while the robot walked on the pebbles surface. The pebbles surface of cobblestones causes slight variations in the load on the sole of the foot with each step, resulting in minor fluctuations in the amplitude of the relative resistance between cycles. By analyzing the response amplitude, waveform shape, and periodic fluctuation characteristics, the cobblestone terrain can still be reliably distinguished, providing a potential sensory basis for robotic ground surface perception and terrain-adaptive locomotion.

Collectively, these static and dynamic tests demonstrate that the proposed rigid–flexible coupled pressure sensor can reliably capture distinct resistance signals characteristic of different ground surfaces. By relying on these responded signatures, including the baseline offset, peak-to-valley amplitude, and fluctuation patterns, the sensor effectively differentiates various types of terrain in a terrain-dependent manner, confirming its good potential for environmental perception in robotic applications. The successful discrimination of four distinct terrains using a single compact sensor element highlights the advantages of our multistructural design, which provides sufficient sensitivity and stability to resolve subtle mechanical differences in the external environment. This capability could be particularly valuable for autonomous robots operating in unstructured settings, where accurate terrain recognition is essential for adaptive locomotion control and navigation decision-making.

## 4. Conclusions

In summary, based on a strategy of coupled bionics, this paper successfully developed a multistructure coupled pressure sensor inspired by the rigid–flexible coupled structure of *Bambusa textilis* and the micro-protrusions of *Salvia plebeia* R.Br., enabling the device to combine rapid recovery with high stability. This sensor features a layered structure consisting of an aluminum substrate, a conductive porous sponge, and a micro-embossed conductive film. The sensing mechanism relies on a multiscale synergistic piezoresistive effect; at low loads, the sponge’s three-dimensional skeleton deforms elastically for sensitive weak-signal detection. As the load increases, the pores progressively collapse, reducing resistance, while the micro-protruded film enhances contact with the sponge, enlarging the conductive area and effectively postponing saturation. This synergy enables a wide detection range of 0–31.5 kPa, and within its operating range (0–23.5 kPa), the relationship between resistance and pressure exhibits a trend that can be fitted to a quadratic term, with a coefficient of determination of 0.9986. The device achieves a force resolution down to 0.2 N, achieves a minimum response time of 350 ms and exhibits stable dynamic tracking across various frequencies. After 5000 loading–unloading cycles, no significant degradation is observed. Mounted on a robotic foot, the sensor successfully discriminates different surfaces with high precision, confirming its practical potential in intelligent environmental perception and flexible robotic applications.

## Data Availability

The original contributions presented in the study are included in the article, further inquiries can be directed to the corresponding author.

## Supplementary Materials

The following supporting information can be downloaded at: https://www.mdpi.com/article/10.3390/biomimetics11080594/s1, Figure S1: Response time comparison; Figure S2: Response variations of the sensor under different pressure.
