# Supplementary material for: A Bioinspired Flexible Pressure Sensor with Rigid–Flexible Coupling Featuring Simple Fabrication, Wide Pressure Range, and High Sensitivity
Source: Biomimetics (Basel). 2026 Aug 20;11(8):594. doi: 10.3390/biomimetics11080594 (PMC13509875; doi:10.3390/biomimetics11080594)
Supplement: Supplementary file 1 [file biomimetics-11-00594-s001.zip › biomimetics-4471001-supplementary.pdf]

Supporting Information

A Bioinspired Flexible Pressure Sensor with Rigid-Flexible Coupling Featuring Simple Fabrication, Wide Pressure Range, and High Sensitivity

Zhen Tang 1, Xingze Chen 2, Xin Wang 2, Yangfan Yang 1, Shanhong Tang 1 and Linpeng Liu 2,*

1 School of Automotive and Energy, Xiangtan Institute of Technology, Xiangtan 411100, China;

15273271107@hngeelyedu.cn (Z.T.); 13549640564@hngeelyedu.cn (Y.Y.);

18773219602@hngeelyedu.cn (S.T.)

2 Sate Key Laboratory of Precision Manufacturing for Extreme Service Performance, College of Mechanical and Electrical Engineering, Central South University, Changsha 410083, China;

253711063@csu.edu.cn (X.C.); 253711004@csu.edu.cn (X.W.)

* Correspondence: linpengliu@csu.edu.cn

Supporting Figures

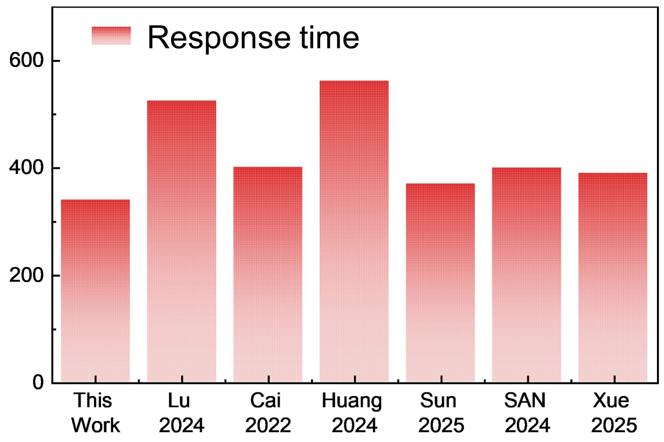

Figure S1. Response time comparison.

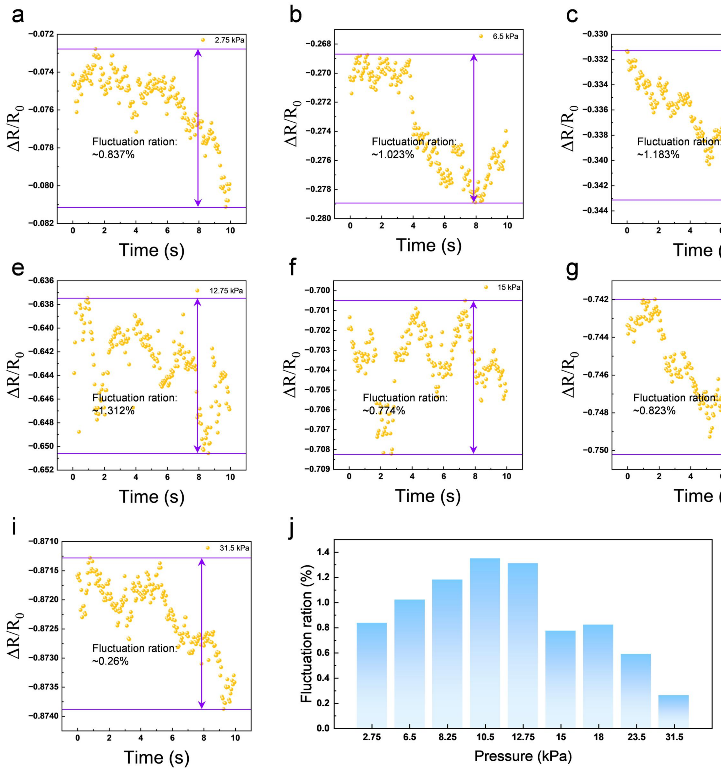

Figure S2. Response variations of the sensor under different pressures. a-i: Relative resistance change, pressure output and resistance drift of the sensor under gradient compression (1 mm per compression step, total compression displacement of 9 mm) for graded compressive strains. g: Signal fluctuation magnitude of sensor resistance under various compressive strain levels.

Reference:

1. Lu, P.; Xu, J.; Wang, X.; Lian, W.; Li, C.; Guan, S. Gradient Pore Structured Ppy/PDMS Conductive Sponge for Flexible Pressure Sensor. *Chem. Eng. J.* **2024**, *488*, 151049, doi:10.1016/j.cej.2024.151049.
2. Cai, Y.; Liu, L.; Meng, X.; Wang, J.; Zhang, C.; Li, J.; Lu, Z.; Duan, J. A Broad Range and Piezoresistive Flexible Pressure Sensor Based on Carbon Nanotube Network Dip-Coated Porous Elastomer Sponge. *RSC Adv.* **2022**, *12*, 34117–34125, doi:10.1039/D2RA06487A.
3. Huang, Q.; Jiang, Y.; Duan, Z.; Wu, Y.; Yuan, Z.; Zhang, M.; Tai, H. Ion Gradient Induced Self-Powered Flexible Pressure Sensor. *Chem. Eng. J.* **2024**, *490*, 151660, doi:10.1016/j.cej.2024.151660.
4. Sun, J.; Li, H.; Ma, X.; Li, Y.; Liu, L.; Song, L.; Wang, X.; Deng, X.; Yu, C.; Zhao, Y. A Dual-Function Flexible Pressure/Humidity Sensitive Sensor Based on a Polyaniline-Coated Melamine Sponge. *New J. Chem.* **2025**, *49*, 9205–9213, doi:10.1039/D5NJ00239G.
5. Srinivasan, R.; Lavanya, J.; Sankar, A.R. Wearable Flexible Pressure Sensor Based on Nitrogen-Functionalized CNT in Melamine Foam for Human Motion Monitoring. *IEEE Access* **2024**, *12*, 194566–194579, doi:10.1109/ACCESS.2024.3518601.
6. Xue, H.; Teng, Y.; Li, J.; Hu, L.; Ding, Y.; Chen, X.; Wang, H.; Li, F.; Zhao, H.; Lin, X.; et al. Resistance-Tuned Electrode Strategy for Achieving an Ultra-Wide Linear Range in Iontronic Pressure Sensors. *IEEE Electron Device Lett.* **2025**, *46*, 1389–1392, doi:10.1109/LED.2025.3581987.
